# Long non‐coding RNA NORAD promotes the prostate cancer cell extracellular vesicle release via microRNA-541-3p-regulated PKM2 to induce bone metastasis of prostate cancer

**DOI:** 10.1186/s13046-021-01891-0

**Published:** 2021-03-16

**Authors:** Chuan-yi Hu, Juan Chen, Xin-hua Qin, Pan You, Jie Ma, Jing Zhang, He Zhang, Ji-dong Xu

**Affiliations:** 1 Department of Urology, Gongli Hospital of Shanghai Pudong New Area, No. 219, Miaopu Road, Pudong New Area, 200135 Shanghai, P.R. China; 2 Department of Gynecology, Gongli Hospital of Shanghai Pudong New Area, 200135 Shanghai, P.R. China; 3 Graduate School, Ningxia Medical University, 750004 Yinchuan, P.R. China

**Keywords:** NORAD, miR-541-3p, Pyruvate kinase isozymes M2, Extracellular vesicles, Prostate cancer, Bone metastasis

## Abstract

**Background:**

Bone metastasis is the leading cause of mortality and reduced quality of life in patients with metastatic prostate cancer (PCa). Long non-coding RNA activated by DNA damage (NORAD) has been observed to have an abnormal expression in various cancers. This article aimed to explore the molecular mechanism underlying the regulatory role of NORAD in bone metastasis of PCa.

**Methods:**

NORAD expression in clinical PCa tissues and cell lines was detected with the application of qRT-PCR. Cancer cells were then transfected with plasmids expressing NORAD, after which Transwell assay and CCK-8 assay were carried out to detect proliferation, migration, and bone metastasis of PCa. NORAD downstream target molecules were screened through bioinformatics analysis, followed by further verification using dual luciferase assay. Extracellular vesicles (EVs) were labeled with PKH67 and interacted with bone marrow stromal cells. The gain- and loss-function method was applied to determine the internalization and secretion of PCa cells-derived EVs under the intervention of downstream target molecules or NORAD.

**Results:**

PCa tissues and cell lines were observed to have a high expression of NORAD, particularly in tissues with bone metastasis. NORAD knockdown resulted in reduced secretion and internalization of EVs, and suppressed proliferation, migration, and bone metastasis of PCa cells. It was indicated that NORAD interacted with miR-541-3p, leading to the upregulation of PKM2. Forced expression of PKM2 promoted the transfer of PKH67-labeled EVs to bone marrow stromal cells.

**Conclusions:**

NORAD might serve as a ceRNA of miR-541-3p to promote PKM2 expression, thereby enhancing the development of bone metastasis in PCa by promoting internalization and transfer of EVs of cancer cells, providing an insight into a novel treatment for the disorder.

## Background

Prostate cancer (PCa) is the most common cancer in men. Although the progression of PCa is usually very slow, it remains to be the third leading cause of cancer related death in men [1]. In Asian countries, PCa is prevalent in patients over the age of 65 years, with its incidence found to have tripled in patients over 70 years of age [2]. Moreover, the risk factors of PCa include sedentary lifestyle, environmental carcinogens, family history of PCa, and oxidative stress associated with the aging process [3]. If diagnosed early, PCa patients can receive surgery and radiation therapy; however, these interventions haven’t been successful in prolonging the 5-year survival of PCa in patients with advanced disease, which remains to be very low [4, 5]. Bone metastasis is the third most common metastatic site of numerous solid tumors including lung cancer, breast cancer, PCa, colorectal cancer, thyroid cancer and melanoma; in the case of PCa, 70% patients develop bone metastasis [6]. Bone metastasis is the main cause that accounts for the reduced quality of life and mortality in patients with metastatic PCa [7]. Reportedly, extracellular vesicles (EVs) released from tumor cells, carrying lncRNA and other bioactive components [8], could potentially modify the bone microenvironment, thereby aiding the formation of bone metastases [9]. Long non-coding RNAs (lncRNAs) have been highlighted as participants in the development of bone metastasis in PCa [10].

Long noncoding RNA activated by DNA damage (NORAD), a recently identified lncRNA, has been observed to be highly expressed in PCa cell lines and promotes the proliferation and migration of PCa cells [11]. However, the underlying mechanism through which NORAD is involved in the progression of PCa remains unknown. Chromosomal abnormalities are present in approximately 60%-80% of human tumors [12], and NORAD is vital in maintaining chromosomal stability and normal mitosis in human bodies [13], which further highlights the role of NORAD in regulating tumor development. The high expression of NORAD has been previously linked with enhanced metastatic potential and clinical progression of bladder cancer, resulting in poor prognosis [14]. It has been well established that lncRNAs compete with specific mRNAs to bind miRNAs [15]. In this study, StarBase was applied, the results of which predicted that there was a binding site for NORAD and miR-541-3p. A recent study revealed that miR-541-3p is involved in regulating the proliferation and cell cycle of PCa cells [16]. Furthermore, miR-541-3p promotes the osteogenic differentiation of human bone mesenchymal stem cells [17], suggesting that miR-541-3p plays a role in bone formation.

In this study, we found that the interaction between NORAD and miR-541-3p promoted bone metastasis by increasing the expression of pyruvate kinase isozymes M2 (PKM2) in PCa cells and their EVs. Highly expressed PKM2 promoted the release of EVs from PCa cells, resulting in the enhancement of the internalization of EVs by bone marrow stromal cells. Based on these findings, it can be concluded that NORAD/PKM2/miR-541-3p axis could serve as a potential therapeutic target for PCa.

## Methods

### Ethical statement

This study was approved and reviewed by the Medical Ethics Committee of Gongli Hospital of Shanghai Pudong New Area, and was conducted in accordance with the Helsinki Declaration. Written informed consent was obtained from all participants prior to the study. All mouse experiments were approved by the Animal Protection and Use Committee of Gongli Hospital of Shanghai Pudong New Area.

### **Bioinformatics analysis**

Downstream miRNAs of NORAD were analyzed with the use of starBase databases (http://starbase.sysu.edu.cn/). Potential target genes of miRNAs were predicted by the intersection of three databases, including starBase (clipExpNum > 20), mirDIP (Score Class, Very High and High, http://ophid.utoronto.ca/mirDIP/), and miRWalk (energy < -25, http://mirwalk.umm.uni-heidelberg.de/). Protein-protein interaction (PPI) networks of hub genes were constructed by String (minimum required interaction score, 0.15, https://string-db.org/) and the screened hub genes were further analyzed in PCa-related microarray dataset GSE38241. The top 10 genes interacting with PKM2 were predicted by String, and KOBAS3.0 (http://kobas.cbi.pku.edu.cn/kobas3) was used to perform Gene Ontology (GO) and KEGG enrichment analysis to predict PKM2 function. Cytoscape (https://cytoscape.org/) was used to visualize PPI and show the degree of network modules.

### **Clinical tissue sample**

Seventy-four PCa tissues and their matched normal tissue samples (2 cm away from cancer tissues) frozen in fresh liquid nitrogen stored in Gongli Hospital of Shanghai Pudong New Area from 2011 to 2015 were collected. The PCa tissue samples were collected from 74 PCa patients (with the age ranging from 55 to 84 years-old) included 25 non-bone metastasis PCa tissues and 49 bone metastasis PCa tissues. All tissue specimens were pathologically confirmed PCa, and the patients did not receive radiotherapy or chemotherapy before surgery. All tissue specimens received a wash with normal saline and were immediately placed into liquid nitrogen for long-term storage. Follow up was continued for 60 months, and the overall survival rate was observed.

### Cell culture

The PCa cell lines (22Rv1, DU145, and PC-3, Shanghai Institute of Life Sciences, Chinese Academy of Sciences) were cultured in RPMI-1640 (Gibco, Carlsbad, California, USA) medium, immortalized prostate epithelial cell line RWPE-1 (Shanghai Institute of Life Sciences) in K-SFM medium (Gibco), C4-2B cell line (MD Anderson Cancer Center, USA) in T medium [80% dulbecco’s modified eagle medium (DMEM, Thermo Fisher Scientific Inc., Waltham, Massachusetts, USA), 20% F12 (Invitrogen, Carlsbad, California, USA), T-medium supplement (Sigma-Aldrich, SF, CA, USA)], human bone marrow stromal cell line HS-5 (Bnbio organisms, Beijing, China) and human embryonic kidney cells HEK293T (American Type Culture Collection (ATCC), VA, USA) in DMEM medium. All medium contained 10% fetal bovine serum (FBS) (Gibco) and 100 ug/mL streptomycin/penicillin (HyClone Company, Logan, UT, USA). All cells were cultured at 37 °C, 5% CO_2_, and 95% saturated humidity. The medium was changed 3–4 times a week depending on the cell growth and the cells were passaged at 80% of confluence.

### Extraction and identification of EVs

EVs in FBS were depleted by centrifugation for 55 min at 3000 g in Amicon ultra-15 centrifugal filters (UFC910024, 100 kDa Merk Millipore Ltd., Tullagreen, Carrigtwohill, Co.Cork, Ireland). PCa cells were cultivated in normal medium to 80–90% confluence, and cultured with EVs-depleted FBS medium. Then, cell culture supernatant was collected and underwent centrifugation at 300 g for 10 min, 2000 g for 10 min, 10,000 g for 30 min, and 100,000 g for 70 min. Next, the precipitates were re-suspended in PBS and precipitated to separate EVs using the ExoQuick-TC™ kit (SBI, CA, USA). EVs were incubated with CM and ExoQuick™ reagent overnight at 4℃, centrifuged at 1500 g for 30 min, suspended in 100 µl PBS and stored at -80℃. Finally, EVs were observed under a transmission electron microscopy (Hitachi H-7650, Tokyo, Japan), during which time Nanoparticle tracking analysis (NTA) (Malvern Instruments, Malvern, UK) was used to detect EV size distribution and concentration, and Western blot to detect expression of EV marker CD9, CD63 and Alix.

### EV uptake

EVs were labeled with 2.5 mM PKH67 green fluorescent dye using PKH67 Green Fluorescent Cell Linker Midi Kit (Sigma-Aldrich) in 400 ml diluent C for 5 min, blocked with 1% bovine serum albumin (BSA) for 1 min, and then washed with PBS at 120,000 g for 2 h at 48℃. Subsequently, PKH67-labeled EVs were resuspended in PBS and stored at -80℃. A total of 1.5 × 10^5^ HS-5 cells were incubated with 4 × 10^6^ PKH67-labeled EVs in 96-well plates for 18 h, or treated with V-ATPase inhibitor [Bafilomycin A1, # 54,645, Cell Signaling Technologies (CST), Beverly, MA, USA]. After undergoing staining with DAPI, the uptake of EVs was observed under a confocal microscope (LSM780, Carl Zeiss MicroImaging, Inc., Thornwood, NY, USA).

### Cell transfection

The cells were transfected using Lipofectamine 3000 (Invitgen, Carlsbad, CA) when reaching 70% confluence according to the instructions. The miR-541-3p mimic, mimic NC, miR-541-3p inhibitor, inhibitor NC, miR-541-3p antagomir, and antagomir NC were all purchased from RiboBio (Guangzhou, China). Transfection reagent and mimic/inhibitor/antagomir were diluted in Opti-MEM (Gibco), left to stand for 15 min, and added to the cell culture medium. Specific short hairpin RNA against NORAD (sh-NORAD), overexpressed NORAD (OE-NORAD), OE-PKM2 were all designed and packaged into lentiviral vectors (Genecopoeia, Rockville, MD, USA). The cells were transfected with lentiviral vectors (MOI = 50), while 5 µg/mL Polybrene (H8761, Beijing Solarbio Science & Technology Co. Ltd., Beijing, China) was added to improve the infection efficiency. Cells were transfected with sh-NORAD, OE-NORAD, OE-PMK2, miR-541-3p inhibitor, miR-541-3p antagomir and corresponding controls alone or in combination.

### Quantitative reverse transcription PCR (qRT-PCR)

Total RNAs were extracted with Trizol reagent (Invitrogen), and EV miRNAs were isolated using SeraMir Exosome RNA Purification Kit (System Biosciences, Mountain View, USA). The transcriptor First Strand cDNA Synthesis Kit (Roche, Basel, Switzerland) was used to synthesize cDNA. The qRT-PCR reaction was performed through FastStart Universal SYBR Green Master Mix (Roche, Indianapolis, USA). The relative expression level of miRNA and mRNA was normalized to that of the internal control U6 and β-actin, respectively, using the 2^−ΔΔCt^ method. The experiments were conducted in triplicate. The primers are shown in Table 1.

**Table 1:** Primer sequences for qRT-PCR

Gene name	Primer sequence
NORAD	F: 5’-TGATAGGATACATCTTGGACATGGA-3’
	R: 5’-AACCTAATGAACAAGTCCTGACATACA-3’
miR-541-3p	F: 5’-TGGTGGGCACAGAATCTGGACT-3’
	R: 5’-CAGTGCGTCGTGGAGT-3’
U6	F: 5’-ATTGGAACGATACAGAGAAGATT-3’
	R: 5’-GGAACGCTTCACGAATTTG-3’
PKM2	F: 5’-ATGTCGAAGCCCCATAGTGAA-3’
	R: 5’-TGGGTGGTGAATCAATGTCCA-3’
β-actin	F: 5’-CATGTACGTTGCTATCCAGGC-3’
	R: 5’-CTCCTTAATGTCACGCACGAT-3’

### Western blot

Cells and EVs were lysed in RIPA (P0013B, Beyotime Institute of Biotechnology, Shanghai, China) containing protease inhibitors (A8260, Solarbio). The protein was separated by sodium dodecyl sulphate-polyacrylamide gel electrophoresis (SDS-PAGE) and then transferred to a polyvinylidene fluoride membrane (Invitrolon™ PVDF/Filter Paper Sandwiches, LC2005, Thermo Fisher Scientific, Massachusetts, USA). PHOS-TAG SDS-PAGE (# 193-16711) was used to detect the phosphorylation level of synaptosome-associated protein of 23 kDa (SNAP-23). The membrane was blocked with a blocking solution containing 5% skimmed milk powder, after which incubation was carried out with rabbit antibody at 4℃ overnight, including rabbit anti-SNAP-23 (ab4114, 1:1000, Abcam Inc., Cambridge, MA, USA), rabbit anti-PKM2 (# 4053, 1: 1000, CST), rabbit anti-CD9 (ab92726, 1: 2000, Abcam), rabbit anti-CD63 (ab134045, 1: 5000, Abcam), mouse anti-Alix (# 2171, 1: 1000, CST), rabbit anti-calnexin (# 2679, 1: 1000, CST), and rabbit anti-β-actin (AC026, 1: 50,000, ABclonal Biotech Co., Ltd, Hubei, China). Then, the membrane was incubated with horseradish peroxidase-labeled goat anti-rabbit IgG (ab150077, 1: 1000, Abcam) and goat anti-mouse IgG (ab6728, 1: 2000, Abcam) at room temperature for 1 h. Next, the blots were developed with enhanced chemiluminescence solution (ECL808-25, Biomiga, Inc., San Diego, California) for 1 min at room temperature, and scanned in a chemiluminescence instrument (GE Healthcare, Chicago, Illinois, United States). Semi-quantification of the bands was performed using Image Pro Plus 6.0 (Media Cybernetics, USA) and normalized to β-actin. Results were expressed as the ratio of target protein gray value to β-actin gray value. The test was conducted in triplicate.

### Dual luciferase assay

The NORAD and PKM gene regions containing the miR-541-3p binding site and the complementary sequence mutation site of the seed sequence were artificially synthesized and cloned into the psiCHECK2 (Promega, Beijing, China) vector to construct a luciferase reporter vector. Human embryonic kidney cells HEK293T were seeded in a 24-well cell culture plate, and transfected with miRNA mimic, wild-type (WT) and mutant (MUT) vectors using Lipofectamine 3000. Forty-eight hours after transfection, the luciferase activity was measured using the Dual-Luciferase® Reporter Assay System (E1910, Promega).

#### Immunofluorescence

Cells were fixed with 4% paraformaldehyde (DF0135, Beijing leagene biotech. Co., Ltd, Beijing, China) at 25℃ for 25 min and incubation was carried out with primary antibodies at 4℃ overnight, including rabbit anti-SNAP23 (ab4114, 1: 100, Abcam) and mouse anti-VAMP3 (66488-1- Ig, 1: 100, Proteintech Group, Inc, Wuhan, China). Cells were incubated with secondary antibody at 37℃ for 1 h, including goat anti-rabbit IgG H&L-FITC (# ab6717) and goat anti-mouse IgG H&L-Cy3 (# ab6717). After incubating, cells were stained with DAPI (Beyotime) for 3 min and observed under a Nikon A1R confocal microscope.

### Adenosine triphosphate (ATP) determination

ATP determination was performed using a previously described method [18]. The initial concentration of EVs was 0.4 mg/ml. ATP ladders was prepared in eight 1: 2 diluted standard reaction buffers, ranging 5 to 0.05 µm, and eight 1: 2 diluted standard reaction buffers, ranging from 10 to 0.1 µm, and was mixed with 10 µl EVs with or without 3.3 mM vanadate at 37℃ for 10 min. ATP levels were determined in accordance with the general framework of ATP determination.

### Cell Counting Kit-8 (CCK-8) assays

PCa cell proliferation was evaluated using CCK-8 (CK04, Tokyo, Japan). Briefly, 5 × 10^3^ PCa cells were cultured in a 96-well plate, and incubated for 1, 2, 3, 4, and 5 days respectively. Subsequently, 10 µl of CCK-8 reagent was added for further culture at 37 ℃ for 2 h. The absorbance of each well was analyzed at 450 nm with an enzyme immunoassay analyzer.

### Transwell assays

Invasion and migration assays were performed in an 8 µm Transwell chamber (Corning, N.Y., USA) with or without Matrigel coating (BD Biosciences, Franklin Lakes, NJ, USA) on the membrane. PCa cells were suspended in serum-free medium at a density of 1 × 10^5^ cells/ml, after which 100 µl of cell suspension was added to the upper chamber of the Transwell chamber. The lower chamber contained 500 µl of complete medium or HS-5 cell conditioned medium (HS-5-CM) or cell suspension containing HS-5 cells. After 24–48 h culture, the cells were fixed with 4% paraformaldehyde. The cells on the upper surface of the membrane were scraped with a cotton swab, and the cells on the lower surface were stained with crystal violet. Finally, five fields were randomly selected and the cells were observed under an optical microscope (IX71; Leitz, Witzlar, Germany).

### Fluorescence in situ hybridization (FISH)

The location of FOXD2-AS1 in PCa cells was detected with the use of FISH, according to instructions of RiboTM lncRNA FISH Probe Mix (Red) (Ribobio Biological Technology Co., LTD, Guangzhou, China). NORAD probe was customized according to NORAD. Briefly, PCa cells were inoculated on a cover slip in a 6-well plate and were cultured for 1 d until the cells reached about 80% confluence. The slide was removed, fixed with 4% paraformaldehyde at room temperature, treated with proteinase K (2 µg/mL), glycine and acetalization reagent, and incubated with 250 µL of prehybridization solution at 42℃ for 1 h. Next, prehybridization solution was aspirated. The slide was hybridized with 250 µL of hybridization solution containing probe (300 ng/mL) at 42℃ overnight, stained with 4’,6-diamidino-2-phenylindole (DAPI, 1: 800) diluted with PBST for 5 min, and transferred to 24-well culture plate. The slide was mounted with anti-fluorescence quencher and observed under a fluorescence microscope with 5 different fields selected (Olympus, Tokyo, Japan).

### Animal experiments

For bone metastasis studies, BALB/c-nu mice (5 to 6 week-old, 18 to 20 g, Shanghai SLAC Laboratory Animal Co., Ltd., Shanghai, China) received isoflurane anesthesia, and 1 × 10^5^ PC-3 cells in 100 µl phosphate buffer was injected into their left ventricle. Bone metastasis was monitored by bioluminescence imaging (BLI), and radiographic lesions in the bone were identified on X-rays. The area of osteolytic lesions was measured using a deformed image analysis software (Universal Imaging Corporation, New York, USA), and the total degree of bone destruction of each animal was expressed in square millimeters. The mice were sacrificed forty-five days later, with their tibias obtained for HE staining with HE kit (C0105, Beyotime). Bone metastasis was graded according to the following criteria. 0: no metastasis; 1: bone lesions covered 1/4 bone width; 2: bone lesions involved 1/4 ~ 1/2 bone width; 3: bone lesions exceeded 1/2 ~ 3/4 bone width; 4: bone lesions exceeded 3/4 bone width. The bone metastasis score of each mouse was the sum of bone lesion scores of all extremities. To observe the effect of EVs on bone metastasis, the mice were injected through tail vein with EVs (20 µl per injection, about 2 × 10^9^ EVs) once every other day for 5 days for pretreatment, inoculated with PC-3 cells, and injected with EVs for one week. Bone metastasis was evaluated after 45 days. PKH67-labeled EVs were injected into the tail vein to observe the transfer of EVs to the bone matrix.

### Statistics analysis

The statistical analysis of the research data was performed using SPSS 21.0 (IBM Corp. Armonk, NY, USA). The measurement data were expressed as mean ± standard deviation. Paired *t-*test was used for comparison between cancer tissue and adjacent normal tissue; unpaired *t-*test was used for other two groups; one-way analysis of variance (ANOVA) was used for comparison between multiple groups with Tukey’s post-hoc test; Comparisons between two groups with different time points were performed using two-way ANOVA followed by Bonferroni. The Kaplan-Meier and log-rank test were used to analyze the survival rates. *p* < 0.05 means significant difference.

## Results

### Highly expressed NORAD promoted the proliferation and metastasis of PCa cells

**Fig. 1:**
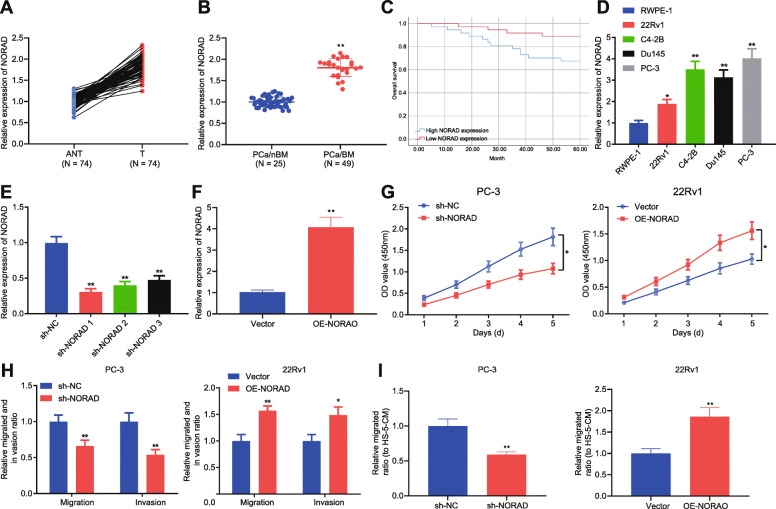
NORAD enhanced the proliferation and metastasis of PCa cells **a**, The expression of NORAD in 74 PCa tissues and adjacent normal tissue was detected by qRT-PCR. * *p* < 0.05, ** *p* < 0.01, vs. adjacent normal tissue. **b**, The expression of NORAD in 25 non-bone metastatic PCa tissues and 49 bone metastasis PCa tissues among 74 PCa patients was detected by qRT-PCR. ** *p* < 0.01, vs. non-bone metastatic PCa tissues. **c**, Kaplan-meier curves of PCa survival (high NORAD, *n* = 37; low NORAD, *n* = 37). **d**, The expression of NORAD in PCa cell lines (22Rv1, C4-2B, Du145, and PC-3) and normal prostate epithelial cell line RWPE-1 was detected by qRT-PCR and the cells with the highest and lowest NORAD expression were selected for follow-up studies. * *p* < 0.05, ** *p* < 0.01, vs. RWPE-1 cells. **e**, The knockdown efficiency of sh-NORAD against NORAD in PC-3 cells was detected by qRT-PCR, and the most efficient sequence was selected for subsequent experiments. ** *p* < 0.01, vs. PC-3 cells treated with sh-NC. **f**, The overexpression efficiency of OE-NORAD against NORAD in 22Rv1 cells was detected by qRT-PCR. ** *p* < 0.01, vs. PC-3 cells treated with vector. **g**, CCK-8 assay of proliferation of PCa cells upon treatment with sh-NORAD or sh-NC. * *p* < 0.05. **h**, Representative images of Transwell assay of migration and invasion of PCa cells upon treatment with sh-NORAD, OE-NORAD or sh-NC. * *p* < 0.05, ** *p* < 0.01, vs. PC-3 cells treated with sh-NC or 22Rv1 cells treated with vector. **i**, The effect of NORAD on the migration of PCa cells towards HS-5-CM was detected by Transwell. ** *p* < 0.01, vs. PC-3 cells treated with sh-NC or 22Rv1 cells treated with vector. The measurement data were expressed as mean ± standard deviation. Paired *t-*test was used for comparison between cancer tissue and adjacent normal tissue; unpaired *t-*test was used for other two groups; ANOVA was used for comparison among multiple groups with Tukey’s post-hoc test; Comparisons between two groups with different time points were performed using two-way ANOVA followed by Bonferroni. The Kaplan-Meier and log-rank test were used to analyze the survival rates. The cell experiment was repeated 3 times

Studies have shown that NORAD induces the proliferation and metastasis of PCa [11], but its regulatory mechanism remains unclear. To provide additional insight on its mechanism, the expression of NORAD was examined in PCa tissues and significantly increased NORAD expression was found in PCa tissues (Fig. 1a), particularly for PCa tissues with bone metastases (Fig. 1b). According to NORAD expression median, patients were divided into high and low expression groups. Kaplan-meier results showed that high expressed NORAD was positively correlated with overall survival of PCa patients (Fig. 1c). It was also found that NORAD was highly expressed in PCa cell lines (22Rv1, C4-2B, DU145, and PC-3) than that in prostate epithelial cell line RWPE-1. Specifically, PC-3 cells had the highest NORAD expression, and 22Rv1 cells had relatively low NORAD expression (Fig. 1d). In order to further study the regulatory effects of NORAD on PCa, NORAD was knocked down in PC-3 cells and overexpressed in 22Rv1 cells (Fig. 1e, f). NORAD knockdown inhibited the proliferation, migration (towards HS-5-CM), and invasion of PCa cells, while NORAD overexpression had the opposite effects (Fig. 1g-i). The above results indicated that NORAD was highly expressed in PCa tissues and cells, and promoted cell proliferation and metastasis.

### NORAD promoted bone metastasis of PCa cells through miR-541-3p

**Fig. 2:**
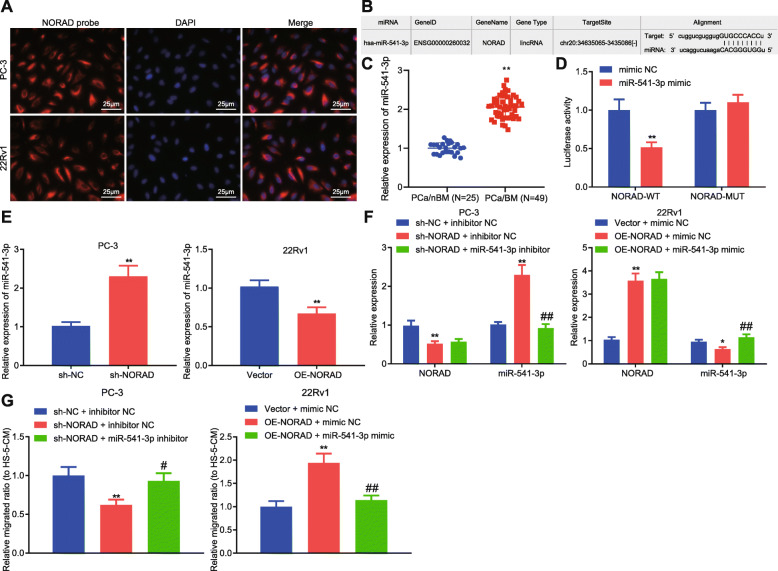
NORAD promoted bone metastasis of PCa cells *in vitro* by inhibiting miR-541-3p **a**, Representative images of FISH with NORAD probe (in red) and DAPI (in blue) in PCa cells (× 400). **b**, Binding site of NORAD and miR-541-3p predicted by StarBase. **c**, The expression of miR-541-3p in PCa tissues detected by PCR. **d**, Dual luciferase assay showed the interaction between NORAD and miR-541-3p, ** *p* < 0.01 vs. HEK293T cells transfected with miR-541-3p mimic NC. **e**, qRT-PCR showed the effect of NORAD on miR-541-3p expression in PCa cells, ** *p* < 0.01 vs. PC-3 cells treated with sh-NC or 22Rv1 cells treated with vector. **f**, qRT-PCR showed NORAD and miR-541-3p expression after treatment with sh-NORAD and miR-541-3p inhibitor, * *p* < 0.05, ** *p* < 0.01 vs. PC-3 cells treated with sh-NC + inhibitor NC or 22Rv1 treated with vector + mimic NC; # *p* < 0.05, ## *p* < 0.05 vs. PC-3 cells treated with sh-NORAD + inhibitor NC or 22Rv1 treated with OE-NORAD + mimic NC. **g**, Transwell showing the effect of NORAD and miR-541-3p on the migration of PCa cells towards HS-5-CM, * *p* < 0.05, ** *p* < 0.01 vs. PC-3 cells treated with sh-NC + inhibitor NC or 22Rv1 treated with vector + mimic NC; # *p* < 0.05, ## *p* < 0.05 vs. PC-3 cells treated with sh-NORAD + inhibitor NC or 22Rv1 treated with OE-NORAD + mimic NC. The measurement data were expressed as mean ± standard deviation. Unpaired *t-*test was used for comparison between two groups. ANOVA was used for comparison between multiple groups with Tukey’s post-hoc test. The cell experiment was repeated 3 times

According to FISH, NORAD was found to be mainly expressed in the cytoplasm (Fig. 2a), suggesting NORAD may act as a ceRNA. NORAD-targeted miR-541-3p was predicted by starBase (Fig. 2b). Additionally, miR-541-3p can result in the inhibition of PCa proliferation by preventing cell cycle progression [16]. As determined by qRT-PCR, miR-541-3p was highly expressed in PCa tissues with bone metastasis (Fig. 2c). The dual luciferase assay also revealed that miR-541-3p overexpression led to the evident reduction of luciferase activity in HEK293T cells transfected with wild-type NORAD (NORAD-WT), but not the mutant NORAD (NORAD-MUT) (Fig. 2d). Moreover, PC-3 cells exhibited increased miR-541-3p secondary to the knockdown of NORAD, and 22Rv1 cells exhibited decreased miR-541-3p after overexpressing NORAD (Fig. 2e). In order to further study the effects of NORAD on bone metastasis of PCa through miR-541-3p, PC-3 cells were transfected with NORAD knockdown and miR-541-3p inhibitor, as 22Rv1 cells with NORAD overexpression and miR-541-3p mimic. The results obtained from qRT-PCR demonstrated that sh-NORAD markedly decreased NORAD expression while increasing miR-541-3p expression; OE-NORAD significantly increased NORAD expression but decreased miR-541-3p expression. Besides, miR-541-3p expression was significantly increased by miR-541-3p mimic (Fig. 2f). Our results from Transwell depicted that PC-3 cells transfected with sh-NORAD reduced migration towards HS-5-CM, which was reversed by miR-541-3p inhibitor; while PC-3 cells transfected with OE-NORAD increased migration towards HS-5-CM, which was reversed by miR-541-3p mimic (Fig. 2g). The results suggested that NORAD can promote the transfer of PCa cells to bone matrix via miR-541-3p downregulation in vitro.

### NORAD promoted the expression of PKM2 in PCa cells and their EVs via miR-541-3p

**Fig. 3:**
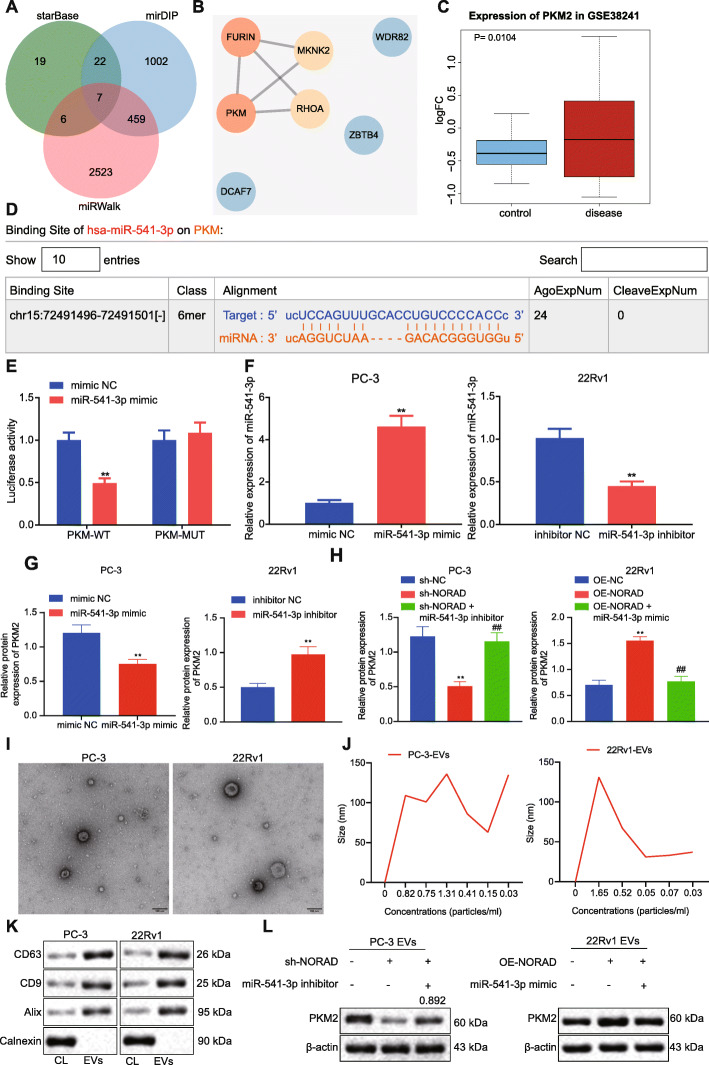
NORAD-targeted miR-541-3p regulated the expression of PKM2 in PCa cells and EVs **a**, Venn diagram of predicted downstream target genes of miR-541-3p through StarBase, mirDIP, and miRWalk. **b**, The PPI of important downstream genes of miR-541-3p. The redder of the circle, the higher the core degree; the bluer of the circle, the lower the core degree. **c**, Box plot of PKM2 expression in microarray dataset GSE38241, with the blue box on the left representing the normal samples and the red box on the right representing PCa samples. **d**, The binding site of miR-541-3p and PKM2 predicted by starBase. **e**, Dual luciferase assay was performed to verify the binding of PKM2 and miR-541-3p, * *p* < 0.05, ** *p* < 0.01 vs. HEK293T treated with mimic NC. **f**, qRT-PCR analysis of the expression of miR-541-3p in PC-3 and 22Rv1 cells, * *p* < 0.05, ** *p* < 0.01 vs. PC-3 cells treated with mimic NC or 22Rv1 cells treated with inhibitor NC. **g**, Western blot analysis of the expression of PKM2 in 22Rv1 treated with miR-541-3p inhibitor and PC-3 cells treated with miR-541-3p mimic. **h**, Western blot analysis of the expression of PKM2 in 22Rv1 treated with OE-NORAD or miR-541-3p mimic and PC-3 cells treated with sh-NORAD or miR-541-3p inhibitor. **i**, Representative images of the morphology of EVs under a transmission electron microscopy (Scale bar, 100 nm). **j**, The size distribution of EVs through NTA. **k**, The expression of specific surface marker protein in EVs was detected by Western blot. **l**, The expression of PKM2 in EVs derived from PCa cells was detected by Western blot. * *p* < 0.05, ** *p* < 0.01 vs. PC-3 cells treated with sh-NC + inhibitor NC or 22Rv1 treated with vector + mimic NC; # *p* < 0.05, ## *p* < 0.05 vs. PC-3 cells treated with sh-NORAD + inhibitor NC or 22Rv1 treated with OE-NORAD + mimic NC. The measurement data were expressed as mean ± standard deviation. Independent t-test was used for comparison between two groups. ANOVA was used for comparison between multiple groups with Tukey’s post-hoc test. The cell experiment was repeated 3 times

The downstream target genes of miR-541-3p via starBase (54 candidates), mirDIP (1490 candidates) and miRWalk (2995 candidates) were predicted to further evaluate the molecular mechanism through which NORAD/miR-541-3p affected PCa. Seven important downstream genes were obtained through taking the intersection (Fig. 3a). PPI network constructed through String demonstrated that of 7 important downstream genes, FURIN and PKM2 (also named PKM in NCBI) revealed the highest degree (Fig. 3b). Through analysis of the microarray dataset GSE38241 in GEO database, we found that PKM2 was a significantly up-regulated gene (*P* = 0.0104) (Fig. 3c), while FURIN was not statistically different between normal tissues and PCa tissues (*P* = 0.111), indicating that PKM2 was a key downstream gene of miR-541-3p. The binding site of miR-541-3p and PKM2 was obtained from starBase (Fig. 3d). The dual luciferase assay confirmed that miR-541-3p could lead to the inhibition of the activity of PKM (Fig. 3e). Additionally, miR-541-3p mimic increased the expression of miR-541-3p and inhibited the expression of PKM2 in PC-3 cells; miR-541-3p inhibitor inhibited the expression of miR-541-3p and increased the expression of PKM2 in 22Rv1 cells (Fig. 3f, g). Similarly, when the expression of NORAD was inhibited in PC-3 cells, the expression of PKM2 was downregulated, and the addition of miR-541-3p inhibitor could relatively restore the expression of PKM2. Overexpressed NORAD up-regulated PKM2; however, miR-541-3p mimic can rescue the expression of PKM2 (Fig. 3h). Reportedly, EVs derived from PCa cells can affect the bone matrix and promote bone metastasis [19]. In order to investigate whether NORAD promotes bone metastasis through PKM2 in EVs from PCa cells, EVs of PCa cells were extracted, identified, and observed under a transmission electron microscope (Fig. 3i). NTA showed that the diameter of EVs was mainly around 30–150 nm (Fig. 3j). Additionally, EVs expressed CD9, CD63 and Alix, rather than the negative marker calnexin (Fig. 3k). When the expression of NORAD was suppressed in PC-3 cells, the expression of PKM2 in EVs was down-regulated; however, miR-541-3p inhibitor relatively restored the expression of PKM2. When NORAD was forced overexpressed in 22Rv1 cells, the expression of PKM2 in EVs was up-regulated, while miR-541-3p mimic rescued the expression of PKM2 in EVs (Fig. 3l). The aforementioned findings suggested that NORAD promoted the expression of PKM2 in PCa cells and EVs through miR-541-3p.

### NORAD promoted the secretion and internalization of PCa cell-derived EVs through miR-541-3p

**Fig. 4:**
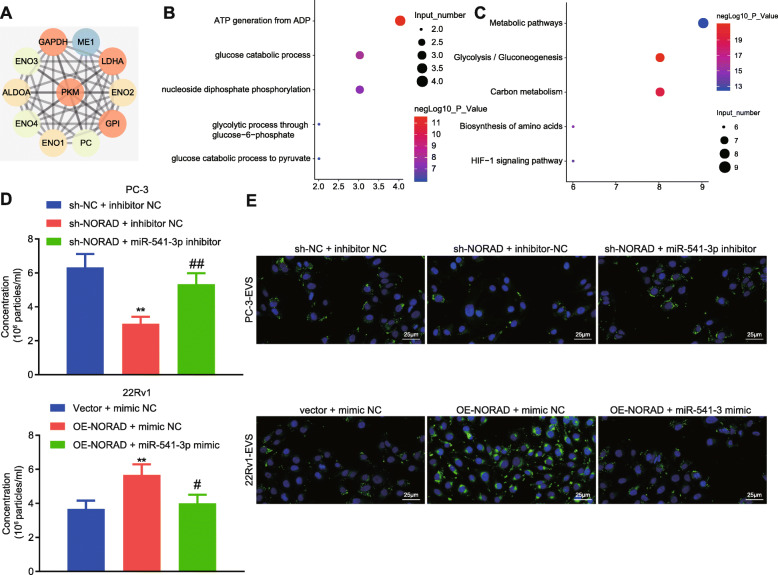
NORAD/miR-541-3p/PKM2 promoted the release and internalization of EVs **a**, Ten center genes interacting with PKM2 from the String PPI network. **b**, Bubble chart showing GO enrichment analysis. Vertical axis represents the top 5 enriched GO terms, the horizontal axis represents the gene number enriched on the GO term, and color (red) intensity represents significance by enrichment p value. **c**, Bubble chart showing KEGG enrichment analysis. Vertical axis represents the top 5 enriched KEGG terms, the horizontal axis represents the gene number enriched on the KEGG term, and color (red) intensity represents significance by enrichment *p* value. **d**, The effect of NORAD and miR-541-3p on the number of EVs in 22Rv1 or PC-3 CM measured by NTA. **e**, Representative images of fluorescence microscope of the internalization of PKH67-labeled EVs (× 400; PKH67, green; DAPI, blue). * *p* < 0.05, ** *p* < 0.01 vs. PC-3 cells treated with sh-NC + inhibitor NC or 22Rv1 cells treated with vector + mimic NC; # *p* < 0.05, ## *p* < 0.01 vs. PC-3 cells treated with sh-NORAD + inhibitor NC or 22Rv1 cells treated with OE-NORAD + mimic NC. The measurement data were expressed as mean ± standard deviation. ANOVA was used for comparison between multiple groups with Tukey’s post-hoc test. The cell experiment was repeated 3 times

In order to determine whether NORAD/miR-541-3p/PKM2 affected bone metastasis of PCa, genes interacting with PKM2 were screened by String, and we got 10 candidate genes (Fig. 4a). Next, these genes were subjected to GO and KEGG enrichment analyses through KOBAS3.0. It was found that the main biological functions of these genes were ATP synthesis and sugar metabolism (Fig. 4b). The pathway analysis revealed significant enrichment in genes involved in metabolic pathway and glycolysis (Fig. 4c). Some studies have shown that EVs-PKM2 of PCa can promote bone metastasis of PCa [20], and that PKM2 affects the release and internalization of EVs [18, 21]. Based on the evidence that NORAD promoted the expression of PKM2 through miR-541-3p, we speculated that NORAD may regulated bone metastasis of PCa via miR-541-3p-targeted PKM2 affecting the release and internalization of EVs. Then, NORAD was downregulated in PC-3 cells, the results of which showed that downregulated NORAD was related to reduced secretion of EVs, and application of miR-541-3p inhibitor rescued the effects of underexpressed NORAD. Similarly, overexpressed NORAD increased the secretion of EVs in 22Rv1 cells, and miR-541-3p mimic rescued the effects of overexpressed NORAD (Fig. 4d). Next, we incubated HS-5 cells with PKH67-labeled EVs and found that fluorescence intensity decreased in HS-5 cells incubated with PC-3-EVs with NORAD silence, indicating reduced internalization of EVs. However, miR-541-3p inhibitor restored the internalization of EVs. The fluorescence intensity enhanced in HS-5 cells incubated with 22Rv1-EVs with NORAD overexpression, indicating enhanced internalization of EVs. miR-541-3p mimic can rescue the effect of overexpressed NORAD (Fig. 4e). Taken together, these results suggested that NORAD interacted with miR-541-3p to promote the release and internalization of EVs and bone metastasis of PCa.

### NORAD promoted the release of EVs through SNAP-23

**Fig. 5:**
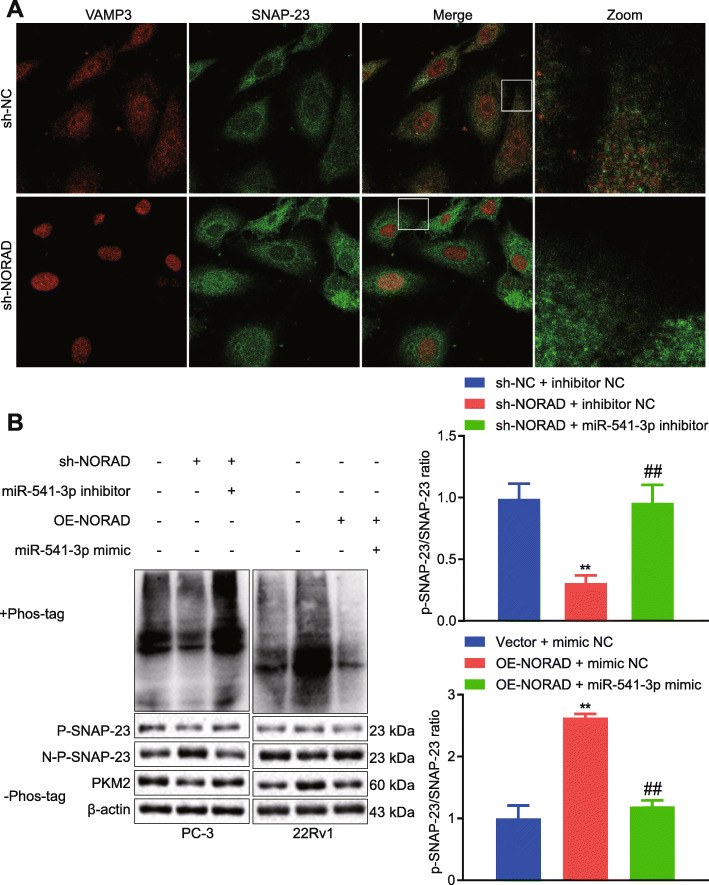
NORAD promoted EVs release via SNAP-23 phosphorylation **a**, Colocalization analysis of VAMP3 (red) and SNAP23 (green) in PC-3 cells with knocked down NORAD. **b**, PKM2 expression and SNAP23 phosphorylation were detected by Western blot in PCa cells with altered expression of NORAD or miR-541-3p. * *p* < 0.05, ** *p* < 0.01 vs. PC-3 cells treated with sh-NC + inhibitor NC or 22Rv1 cells treated with vector + mimic NC; # *p* < 0.05, ## *p* < 0.01 vs. PC-3 cells treated with sh-NORAD + inhibitor NC or 22Rv1 cells treated with OE-NORAD + mimic NC. The measurement data were expressed as mean ± standard deviation. ANOVA was used for comparison between multiple groups with Tukey’s post-hoc test. The cell experiment was repeated 3 times

It has been reported that PKM2 phosphorylates SNAP-23, and then forms a SNARE complex to promote the release of EVs [21]. Therefore, we speculated that NORAD can regulate SNARE complex via miR-541-3p/PKM2 to promote the release of EVs. In PC-3 cells, sh-NORAD reduced the co-localization of VAMP3 and SNAP23, and reduced SNARE complex (Fig. 5a). Additionally, NORAD knockdown in PC-3 cells reduced the expression of PKM2 and phosphorylation of SNAP23; however, miR-541-3p inhibitor rescued the effect of NORAD knockdown. NORAD overexpression in 22Rv1 cells increased the expression of PKM2 and the phosphorylation of SNAP23, however, miR-541-3p mimic rescued the effect of NORAD overexpression (Fig. 5b). These suggested that NORAD can regulate PKM2 through miR-541-3p to promote the phosphorylation of SNAP23, thus inducing the release of EVs.

### NORAD promoted the internalization of EVs by upregulating the production of ATP in EVs

**Fig. 6:**
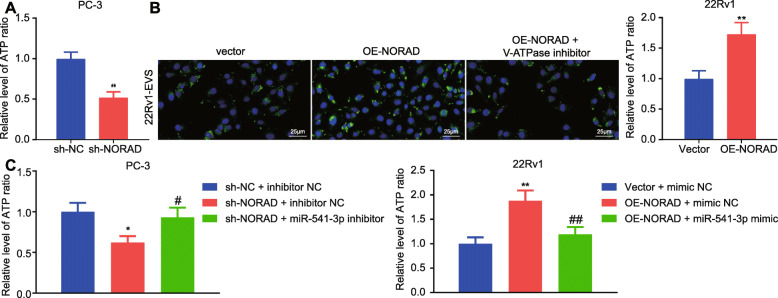
NORAD promoted the production of ATP in EVs **a**, Determination of ATP production in EVs, * *p* < 0.05, ** *p* < 0.01 vs. PC-3 cells treated with sh-NC or 22Rv1 cells treated with vector. **b**, The internalization of EVs was observed with a fluorescent microscope after PKH67-labeled EVs were treated with V-ATPase inhibitors (× 400; PKH67, green; DAPI, blue). **c**, ATP production in EVs was measured, * *p* < 0.05, ** *p* < 0.05 vs. PC-3 cells treated with sh-NC + inhibitor NC or 22Rv1 cells treated with vector + mimic NC; # *p* < 0.05, ## *p* < 0.05 vs. PC-3 cells treated with sh-NORAD + inhibitor NC or 22Rv1 cells treated with OE-NORAD + mimic NC. The measurement data were expressed as mean ± standard deviation. Unpaired *t-*test was used for other two groups; ANOVA was used for comparison between multiple groups with Tukey’s post-hoc test. The cell experiment was repeated 3 times

Next, the role of NORAD/PKM2/miR-541-3p on the internalization of EVs was determined. ATP content was evaluated in EVs, the results of which found that NORAD knockdown in PC-3 cells reduced the ATP content in EVs, and NORAD overexpression in 22Rv1 cells increased the ATP content in EVs (Fig. 6a). Moreover, V-ATPase inhibitors rescued the promoting effects of NORAD overexpression on the internalization of EVs in 22Rv1 cells (Fig. 6b). Additionally, the ATP content decreased in PC-3 cells treated with sh-NORAD + inhibitor NC, and increased in PC-3 cells treated with sh-NORAD + miR-541-3p inhibitor. Besides, the ATP content increased in 22Rv1 cells treated with OE-NORAD + mimic NC, and decreased in 22Rv1 cells treated with OE-NORAD + miR-541-3p mimic (Fig. 6c). Taken together, NORAD promoted the generation of ATP and the internalization of EVs through miR-541-3p/PKM2.

### Promotive effects of NORAD on bone metastasis of PCa cells ***in vivo*** through miR-541-3p/PKM2

**Fig. 7:**
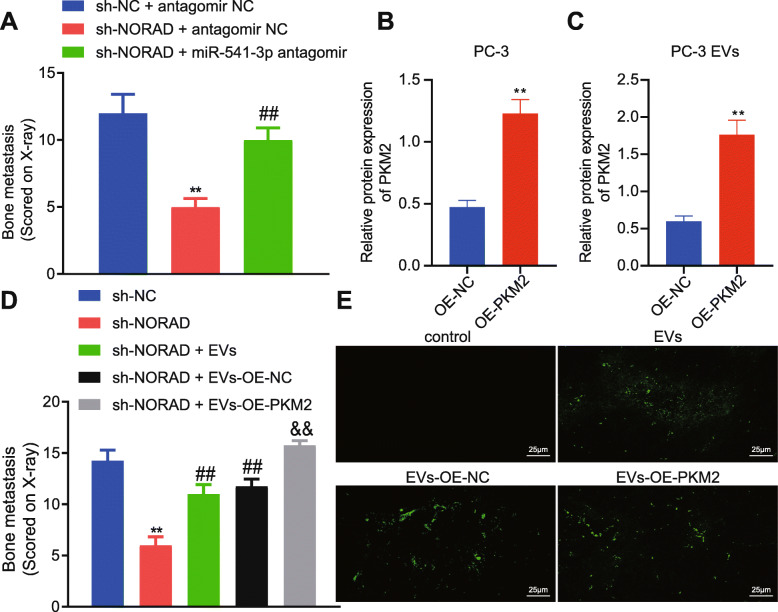
NORAD/miR-541-3p/EVs-PKM2 promoted bone metastasis of PCa cells *in vivo* **a**, The sum of bone metastasis scores of each mouse (*N* = 8). * *p* < 0.05, ** *p* < 0.01 vs. mice treated with sh-NC + antagomir NC; # *p* < 0.05, ## *p* < 0.01 vs. mice treated with sh-NORAD + antagomir NC. **b**, The expression of PKM2 in PC-3 cells transfected with OE-PKM2 was detected by Western blot. **c**, The expression of PKM2 in EVs from PC-3 cells transfected with OE-PKM2 was detected by Western blot. **d**, The sum of bone metastasis scores of each mouse treated with EVs with highly expressed PKM2 (*N* = 8) (× 400). * *p* < 0.05, ** *p* < 0.01 vs. mice treated with sh-NC; # *p* < 0.05, ## *p* < 0.01 vs. mice treated with sh-NORAD; & *p* < 0.05, && *p* < 0.01 vs. mice treated with sh-NORAD + OE-NC. **e**, Representative images of mouse bone marrow 24 hours after intravenous injection of fluorescein-labeled EVs. Scale bar, 25 µm; green, PKH67. The measurement data were expressed as mean ± standard deviation. Unpaired *t-*test was used for other two groups; ANOVA was used for comparison between multiple groups with Tukey’s post-hoc test. The cell experiment was repeated 3 times

A mouse model of bone metastasis was constructed where PC-3 cells with NORAD knockdown or miR-541-3p inhibitor were inoculated into the left ventricle of nude mice in order to determine the effect of NORAD on bone metastasis of PCa *in vivo*. Forty-five days later, we found that NORAD knockdown resulted in reduction of bone metastasis, while miR-541-3p inhibitor alleviated the effects (Fig. 7a). Next, we overexpressed PKM2 in PC-3 cells (Fig. 7b), extracted EVs with highly expressed PKM2 (Fig. 7c), treated the mice with EVs, and observed the effect of EVs on bone metastasis 45 days later (Fig. 7d). NORAD knockdown in PC-3 cells reduced bone metastasis; EVs promoted bone metastasis of PC-3 cells; EVs with highly expressed PKM2 further promoted bone metastasis of PC-3 cells. These findings suggested that PKM2 in tumor EVs can reverse the inhibitory effects of NORAD knockdown on bone metastasis. Finally, fluorescein-labeled EVs were intravenously injected into mice. Twenty-four hours later, the lable-ed EVs were observed in bone marrow stromal cells, and PKM2 overexpression further facilitated the transfer of EVs to bone marrow stromal cells (Fig. 7e). The above results suggested that NORAD can target miR-541-3p to promote bone metastasis of PCa, and this process can be promoted by the increased expression of PKM2 in EVs.

## Discussion

**Fig. 8:**
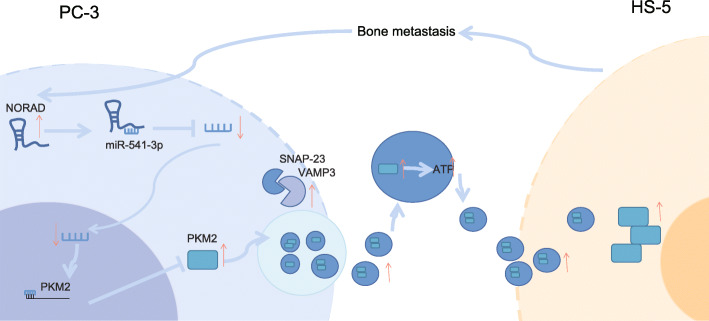
Schematic diagram depicting the proposed molecular mechanism of NORAD in PCa. The expression of NORAD in PCa was high and inhibited miR-541-3p, thereby promoting the expression of PKM2 in PCa cells and PCa cell derived-EVs. The increased PKM2 in PCa cells promoted the phosphorylation of SNAP-23 and formed SNARE complex to promote the release of EVs. The increased PKM2 in EVs promoted the generation of ATP in EVs and the internalization of EVs, thereby affecting the bone matrix and promoting bone metastasis

While the prevalence of PCa continues to rise, the currently available screening or early detection methods remain to be ineffective; in addition, the slow course of the disease coupled with the adverse effects of surgical and radiotherapy, which include uremic symptoms and sexual dysfunction, have made the management of the disease increasingly challenging [22–24]. In addition, metastasis to bones, which has quite a common incidence in PCa, further contributes to the poor prognosis seen in some patients [25]. It’s well known that EVs from PCa cells are enriched in lncRNAs targeting miRNAs [26]. Recently, lncRNAs have been implicated in the development of multiple pathologies, thereby becoming an area of interest for investigators [27, 28]. Our findings revealed the presence of a high expression of NORAD in PCa tissues and cell lines, promoting proliferation, migration, and bone metastasis, all of which were associated with increased secretion of EVs by PCa cells and internalization of EVs. The enhanced secretion and internalization of EVs was mediated by NORAD-promoted PKM2. These data led to the hypothesis that NORAD might serve as a novel therapeutic target for PCa (Fig. 8).

The involvement of lncRNA in the occurrence and development of various diseases, and its role in bone metastasis has been previously highlighted. Li Zhang et al. demonstrated that lncRNA34a regulates bone metastasis in hepatocellular carcinoma [29]. According to Meijuan Liu et al., lncRNA MALAT1 promotes tumorigenesis and bone metastasis in patients with non-small cell lung cancer [30]. Interestingly, NORAD has been proven to be overexpressed in a range of cancers, including pancreatic cancer [31] and bladder cancer [14]; in both cases, high expression of NORAD was associated with poor survival. PCa cell lines have been observed to have highly expressed NORAD, which promotes the proliferation and migration of PCa cells [11]. In the present study, we found highly expressed NORAD in PCa, and that NORAD could promote proliferation and migration of PCa cells.

Bone is the third most common metastatic site for solid tumors, and 70% of patients with metastatic PCa have bone metastases [32]. The metastasis of PCa to the bones severely affects the quality of life in the patients [7]. However, the mechanism regarding bone metastasis is yet to be extensively studied. One of the characteristics of cancer is genomic instability [33], which is associated with metastasis and poor prognosis [34]. Accumulated genomic instability can lead to abnormal metabolism, accelerated aging and cancer development [35–37] while activating NORAD simultaneously [38]. Importantly, NORAD can promote the progression of hepatocellular carcinoma [39], colorectal cancer [40], non-small cell lung cancer [41, 42], and malignant melanoma [43] by targeting different miRNAs. In this study, with the use of starbase, we predicted the binding site between NORAD and miR-541-3p. According to previous data, miR-541-3p inhibits the proliferation of PCa by blocking the cell cycle [16]. Subsequently, PKM2 was identified as the target gene of miR-541-3p through the databases mirDIP, starbase, and mirwalk. There’s increasing evidence suggesting that the activity of PKM2 is essential for the survival of tumor cells [44, 45]. PCa patients were found to have a high expression of PKM2 [46], which promote bone metastasis of PCa [20].

EVs secreted by tumors are the key mediators of communication between tumor cells and distant metastatic organs [47]. Kyoko Hashimoto et al. [48] found that several PCa cell lines release a group of EVs-miRNAs that induce bone sclerosis damage. Non-coding RNA, proteins and other molecules in EVs are well protected from degradation [49]. Our experimental results showed that NORAD could up-regulate the expression of PKM2, resulting in an increase in EVs carried by PKM2. Moreover, the internalization of EVs requires ATP. The internalization of EVs into recipient cells requires energy, and ATP in EVs might play a role in this process [18]. Similarly, a study showed that an increase in PKM2 resulted in alterations in glucose metabolism and promoted the synthesis of ATP [50]. Furthermore, PKM2 can also phosphorylate SNAP-23, thereby promoting the release of EVs [21].

## Conclusion

Collectively, our findings suggested that NORAD was increased in PCa cells and enhanced bone metastasis. NORAD was also found to interact with miR-541-3p, increasing PKM2, thereby promoting the release and internalization of EVs. However, further studies are required to determine the mechanism by which EVs-PKM2 affects bone marrow stromal cells to promote bone metastasis. Our study provided additional insight on the components that make up this fertile tumor metastasis site; nevertheless, their role in tumor growth in bones needs more investigation. Generally, this study provided a new mechanism for bone metastasis of PCa, by showing that targeting NORAD/miR-541-3p/PKM2 might serve as a molecular basis for clinical treatment for PCa patients.

## Data Availability

All data generated or analyzed during this study are included in this article.

## Abbreviations

PCa: Prostate cancer
NORAD: Non-coding RNA activated by DNA damage
EVs: Extracellular vesicles
lncRNAs: Long non-coding RNAs
ATCC: American Type Culture Collection
FBS: Fetal bovine serum
GO: Gene Ontology
CCK-8: Cell Counting Kit-8
HS-5-CM: HS-5 cell conditioned medium
FISH: Fluorescence in situ hybridization
BSA: Bovine serum albumin
CST: Cell Signaling Technologies
sh-NORAD: short hairpin RNA against NORAD
qRT-PCR: Quantitative reverse transcription PCR
SDS-PAGE: Sodium dodecyl sulphate-polyacrylamide gel electrophoresis
SNAP-23: Synaptosome-associated protein of 23 kDa
WT: Wild-type
MUT: Mutant
ATP: Adenosine triphosphate
BLI: Bioluminescence imaging
ANOVA: analysis of variance
